# Supplementary material for: Evaluating cell lines as models for metastatic breast cancer through integrative analysis of genomic data
Source: Nat Commun. 2019 May 15;10:2138. doi: 10.1038/s41467-019-10148-6 (PMC6520398; doi:10.1038/s41467-019-10148-6)
Supplement: Supplementary file 1 — Supplementary Information [file 41467_2019_10148_MOESM1_ESM.pdf]

Evaluating cell lines as models for metastatic breast cancer through integrative analysis of genomic data

Liu et al.

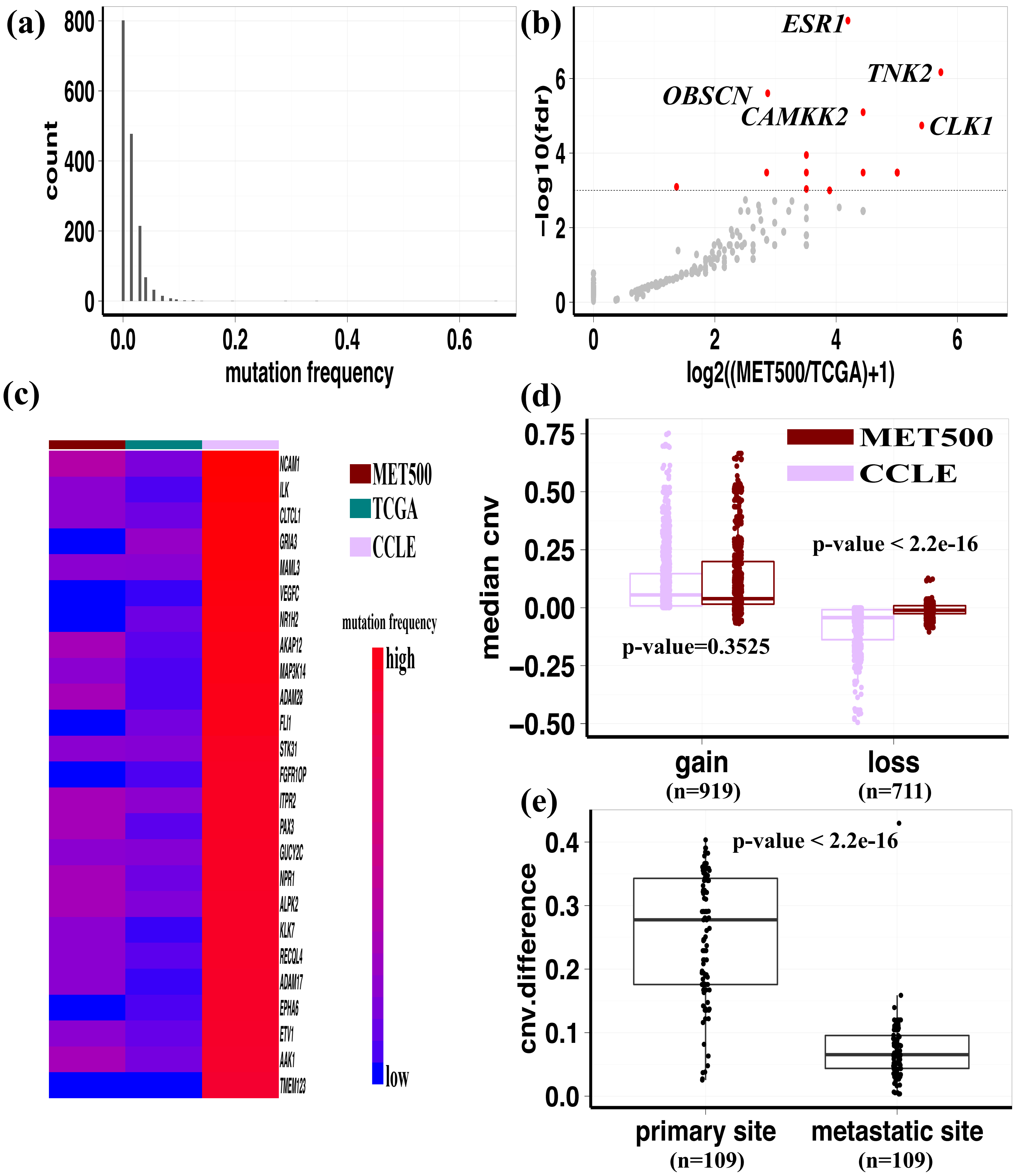

Supplementary Figure 1. (a) Long-tailed gene mutation spectrum in MET500 breast cancer samples. (b) Volcano plot of gene differential mutation analysis. Each dot is a gene, x-axis represents mutation frequency ratio (MET500/TCGA) and y-axis represents FDR (in -log10 scale). The dashed line corresponds to FDR = 0.001. (c) Visualization of log10 transformed mutation frequency of the 25 genes that are specifically hyper-mutated in CCLE breast cancer cell lines. (d) Boxplot of median CNV of grouped genes in MET500 breast cancer samples and CCLE breast cancer cell lines. Genes are grouped according to whether showing gain or loss of copy number in CCLE breast cancer cell lines. (e) CCLE breast cancer cell lines derived from metastatic sites more closely resemble the CNV status of genes with high copy-number-gain in MET500 breast cancer samples. Each dot in the two boxplots represents a gene. (Left: absolute value of median-CNV difference between MET500 breast cancer samples and CCLE breast cancer cell lines derived from primary sites. Right: absolute value of median-CNV difference between MET500 breast cancer samples and CCLE breast cancer cell lines derived from metastatic sites). In panel (d) and (e), P-values are computed with the two-sided Wilcoxn rank sum test. In each box, the central line represents median value and the bounds represent the 25th and 75th percentiles (interquartile range). The whiskers encompass 1.5 times the interquartile range.

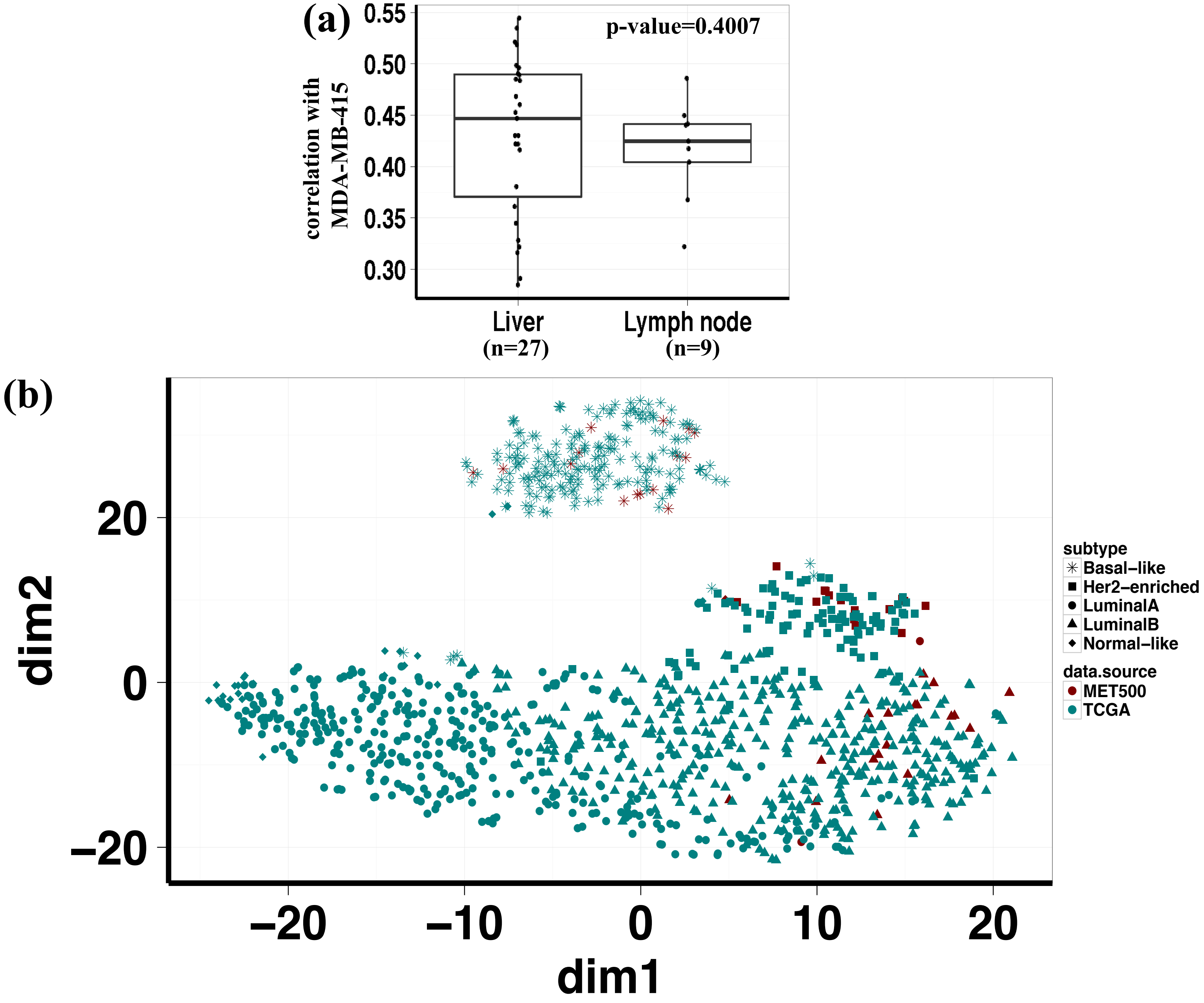

Supplmentary Figure 2. (a) MET500 breast cancer samples derived from liver and lymph node do not show significantly different expression correlation with MDA-MB-415. P-value is computed with the two-sided Wilcoxn rank sum test. In each box, the central line represents median value and the bounds represent the 25th and 75th percentiles (interquartile range). The whiskers encompass 1.5 times the interquartile range. (b) t-SNE plot of TCGA and MET500 breast cancer samples. Data-sources are labeled by color and subtypes are labeled by shape.

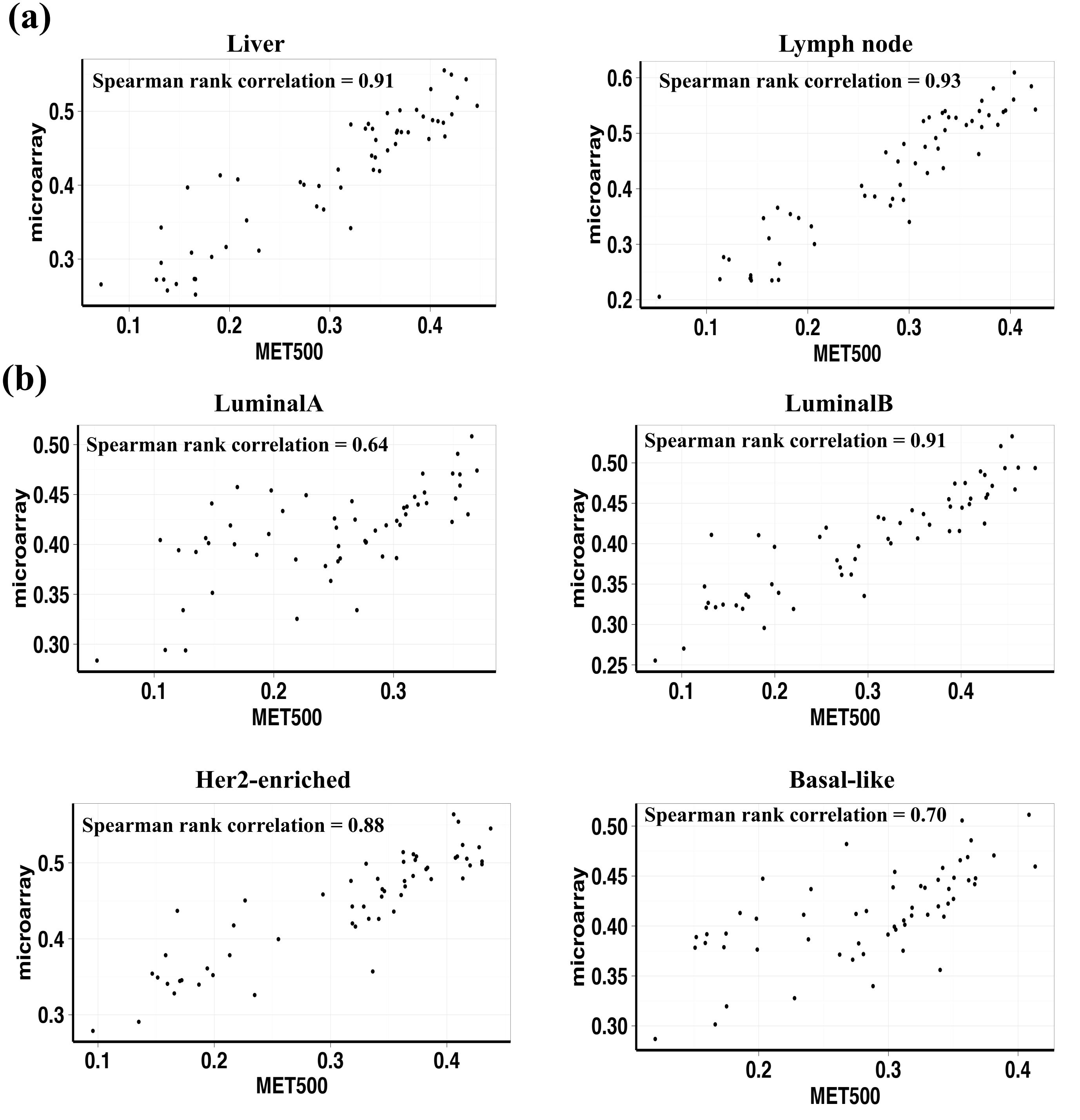

Supplementary Figure 3. (a) Metastatic-site-specific TC analysis results are highly correlated between MET500 dataset and the microarray dataset. (b) Subtype-specific TC analysis results are highly correlated between MET500 dataset and the microarray dataset. In each plot, a dot is a CCLE breast cancer cell line, with x-axis representing transcriptome-similarity derived from MET500 dataset, and y-axis representing transcriptome-similarity derived from the microarray dataset.

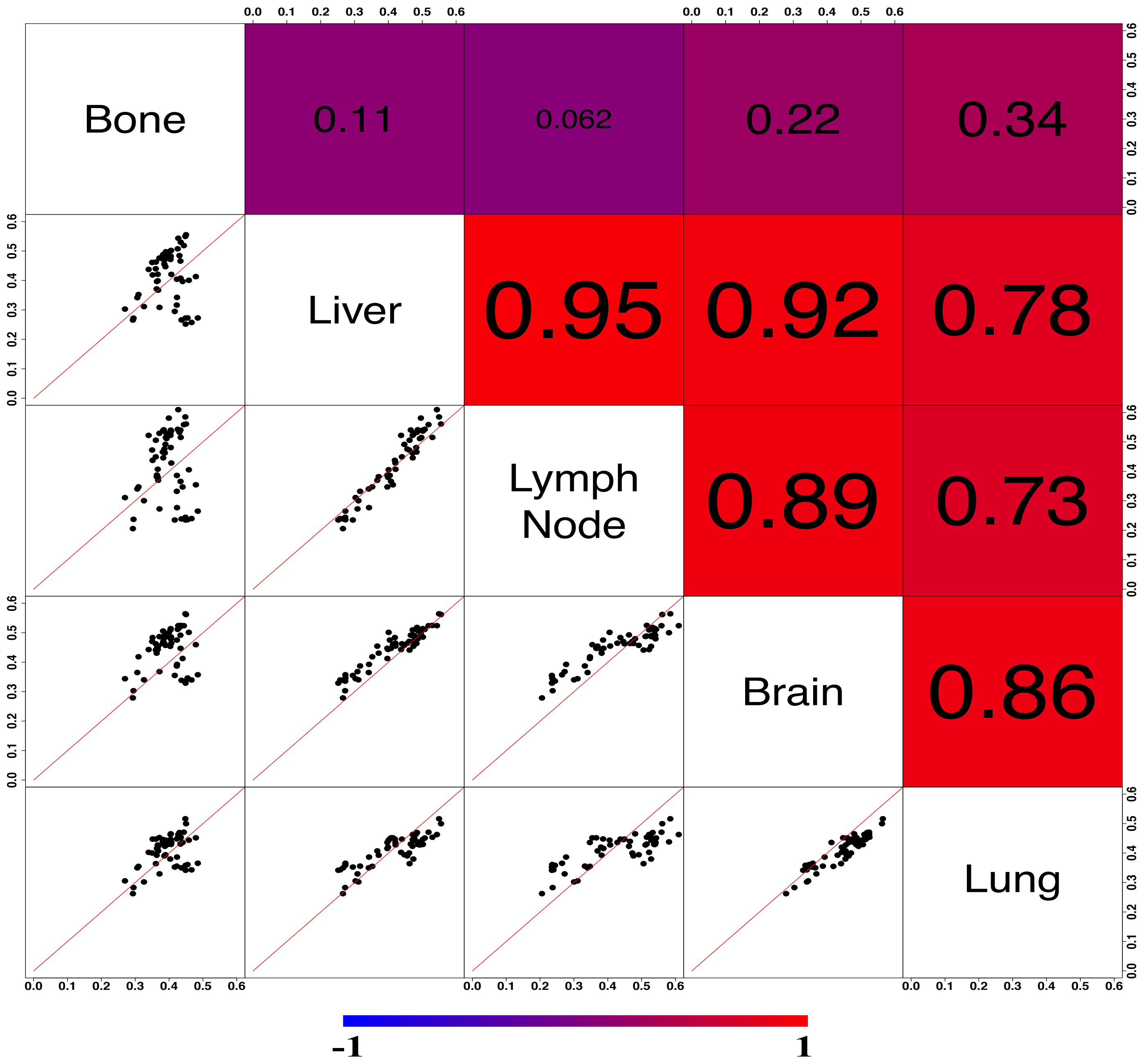

Supplementary Figure 4. Pair-wise comparison of metastatic-site-specific TC analysis results among metastatic sites (microarray dataset).

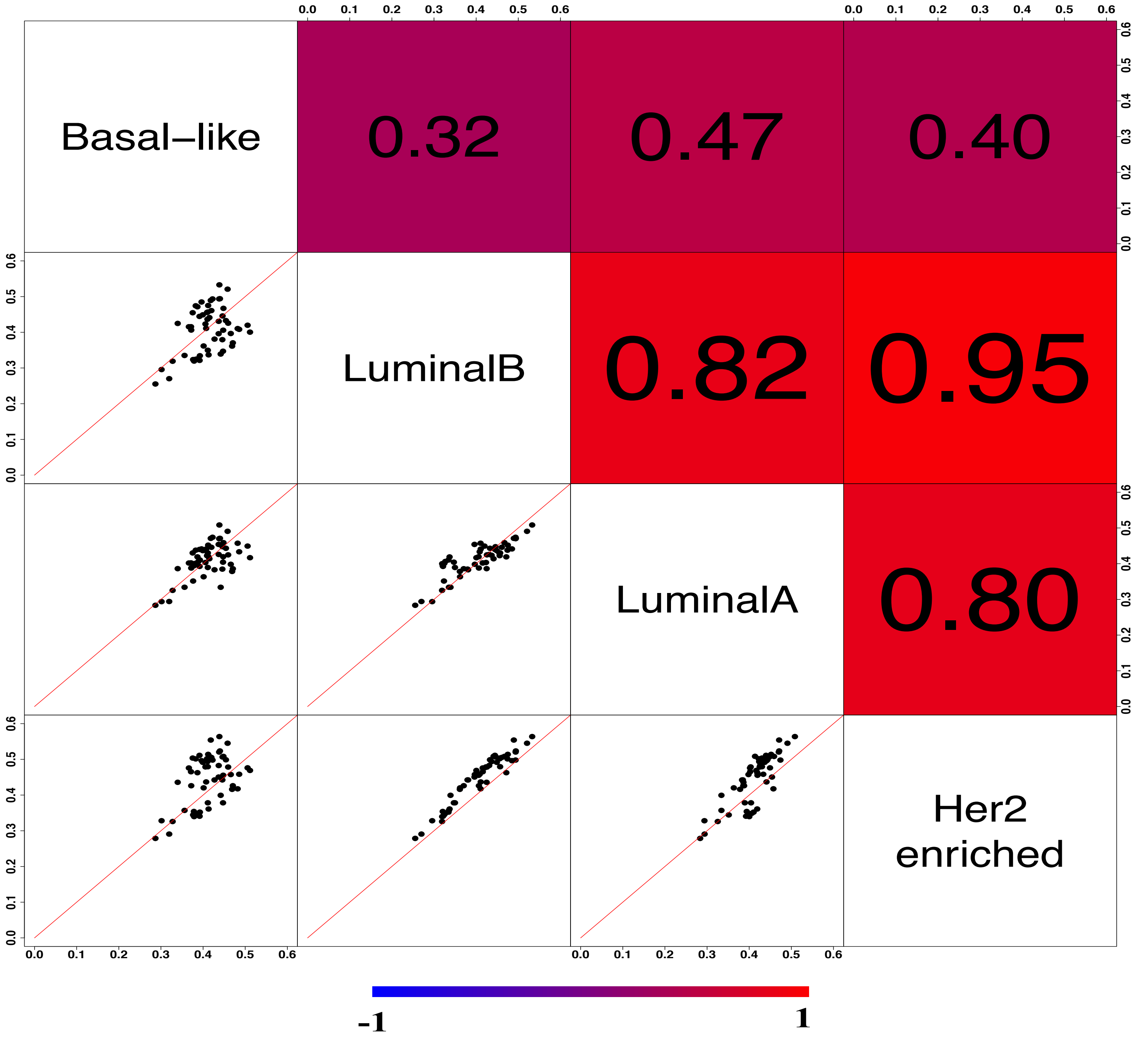

Supplementary Figure 5. Pair-wise comparison of subtype-specific TC analysis results among subtypes (microarray dataset).

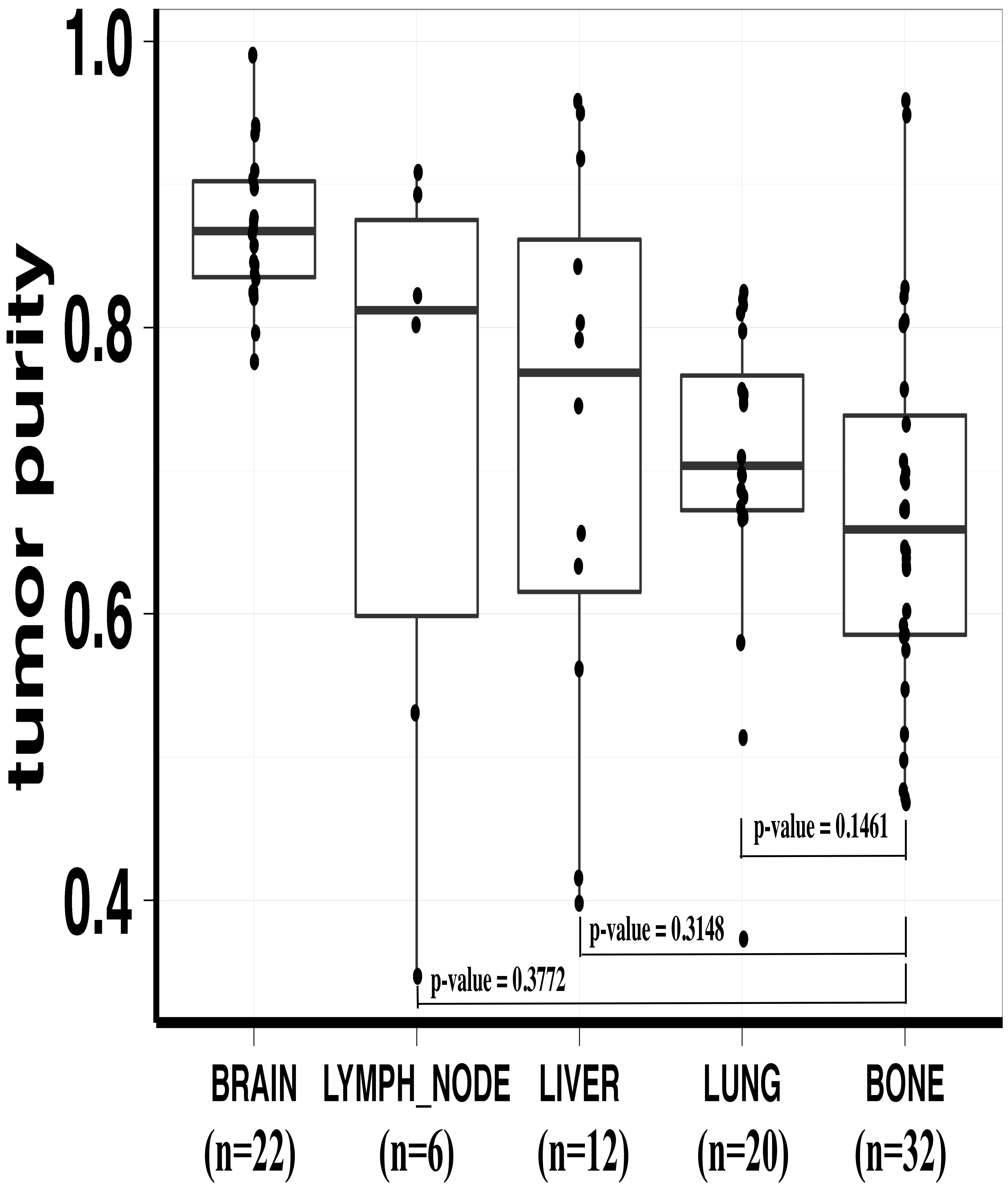

Supplementary Figure 6. Boxplot of tumor purity (microarray dataset). P-values are computed with the two-sided Wilcoxn rank sum test. In each box, the central line represents median value and the bounds represent the 25th and 75th percentiles (interquartile range). The whiskers encompass 1.5 times the interquartile range.

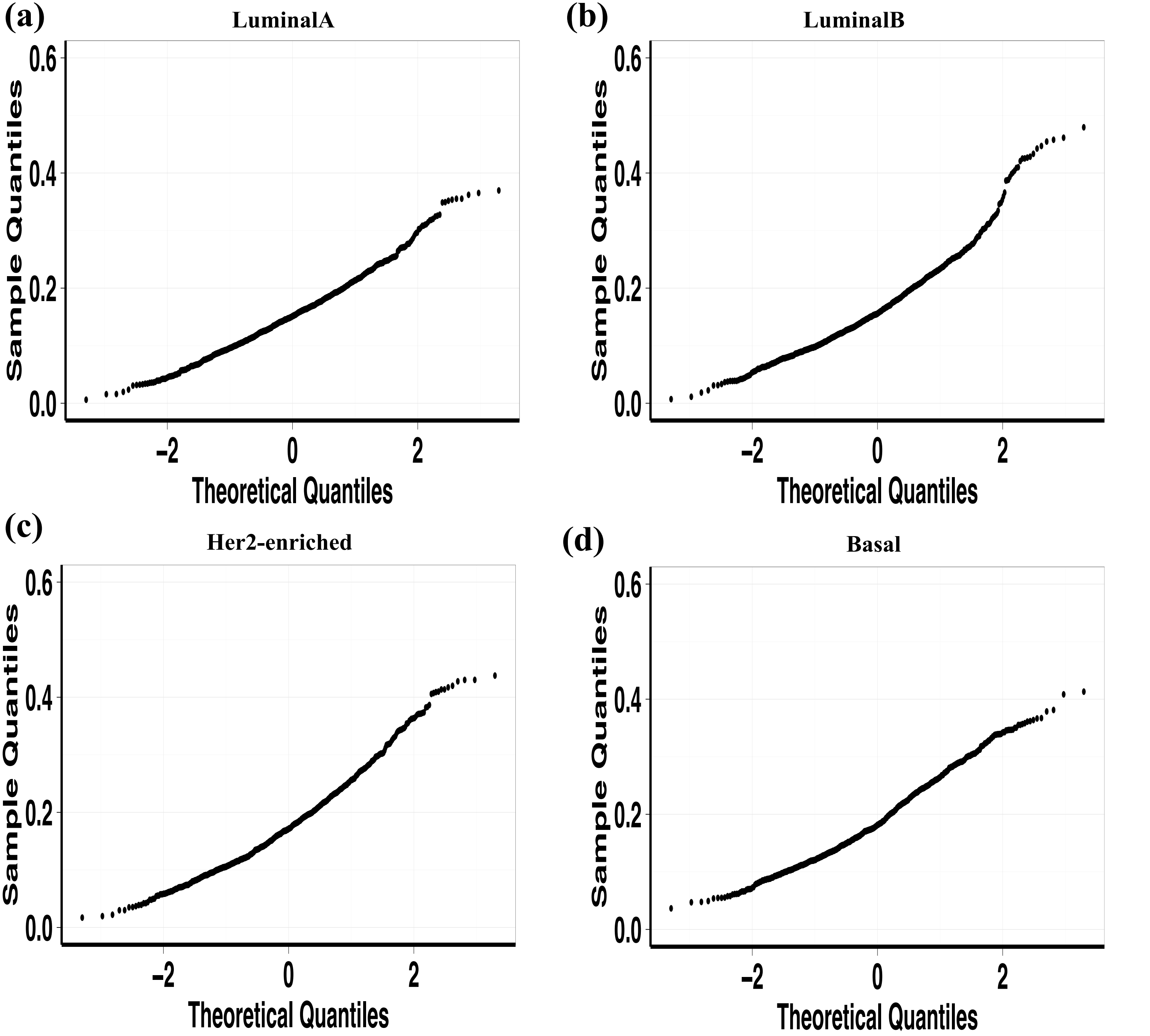

Supplementary Figure 7. Normal QQ-plot to confirm the transcriptome-similarity between a random CCLE cell line and MET500 breast cancer samples of a specific subtype approximately follows normal distribution. (a) LuminalA subtype. (b) LuminalB subtype. (c) Her2-enriched subtype. (d) Basal-like subtype.

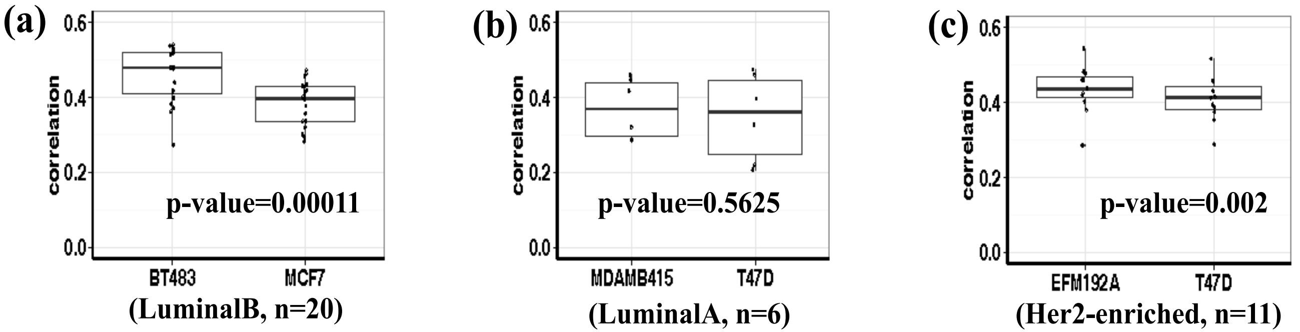

Supplementary Figure 8. (a) Compared to BT483, MCF7 shows significantly lower expression correlation with LuminalB MET500 breast cancer samples. (b) Compared to MDA-MB-415, T47D does not show significantly lower expression correlation with LuminalA MET500 breast cancer samples. (c) Compared to EFM192A, T47D shows significantly lower expression correlation with Her2-enriched MET500 breast cancer samples. In panel (a), (b) and (c), P-values are computed with the two-sided Wilcoxn rank sum test. In each box, the central line represents median value and the bounds represent the 25th and 75th percentiles (interquartile range). The whiskers encompass 1.5 times the interquartile range.

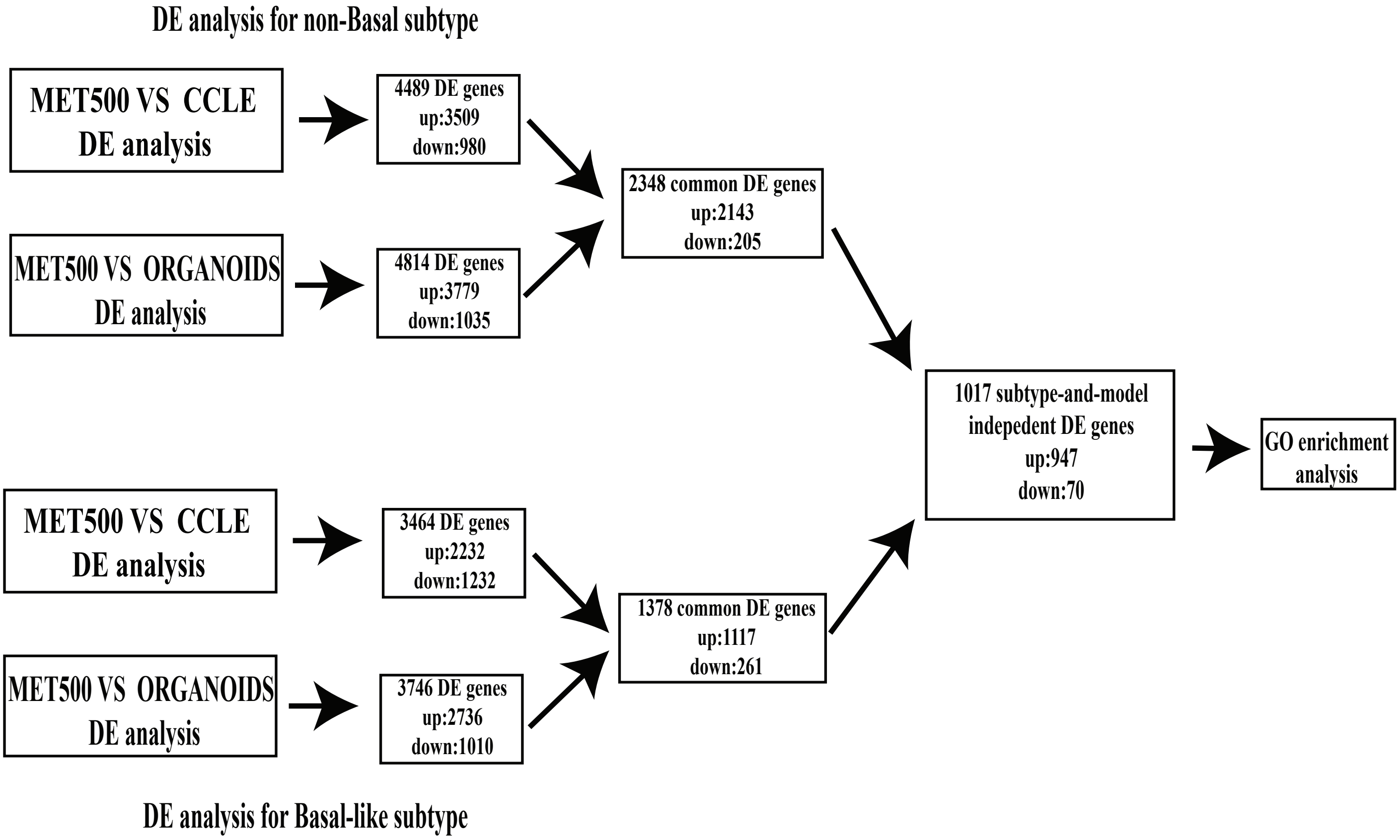

Supplementary Figure 9. Workflow of differential gene expression analysis.

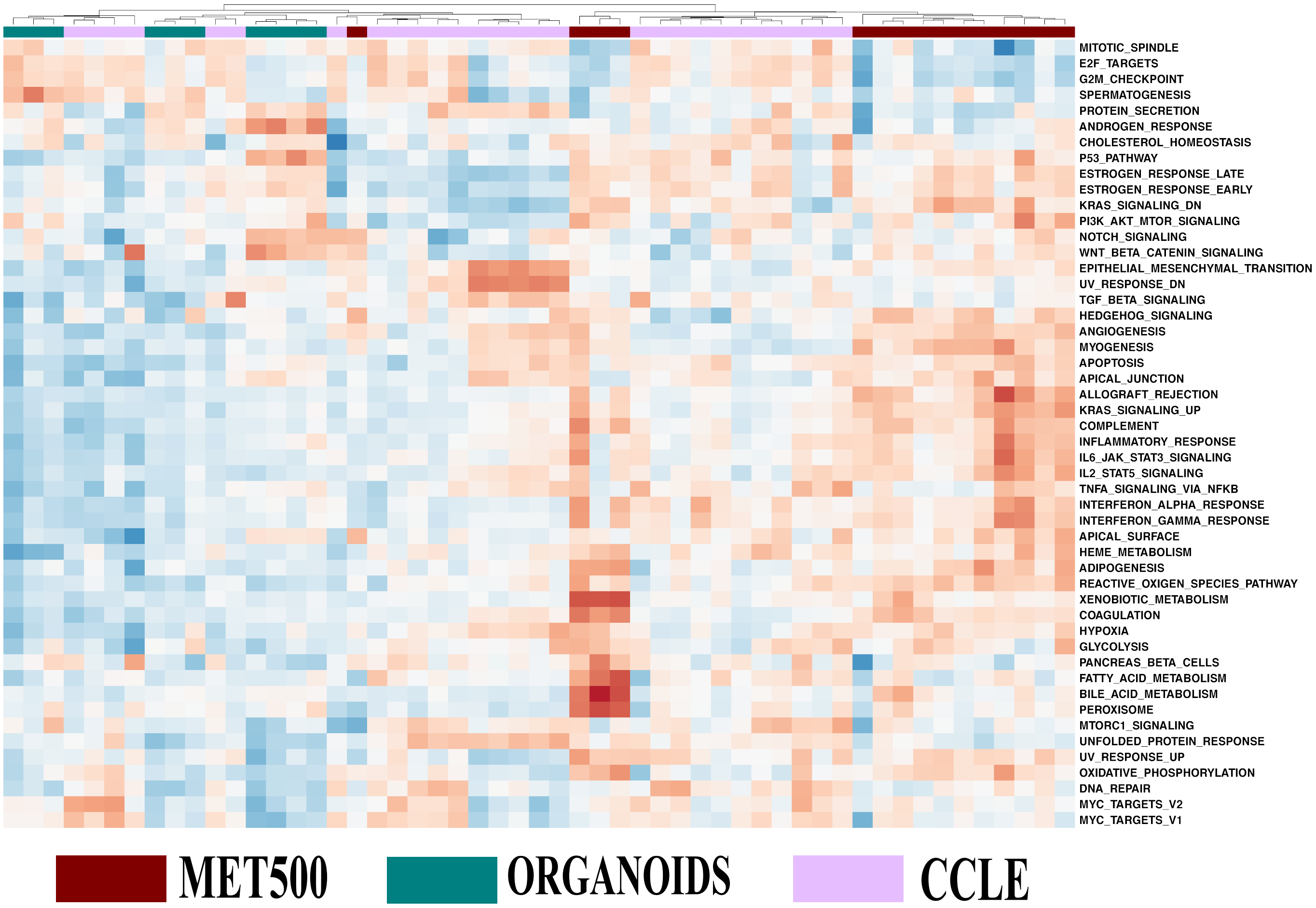

Supplementary Figure 10. Visualization of ssGSEA scores across Basal-like CCLE breast cancer cell lines, MET500 breast cancer samples and organoids.

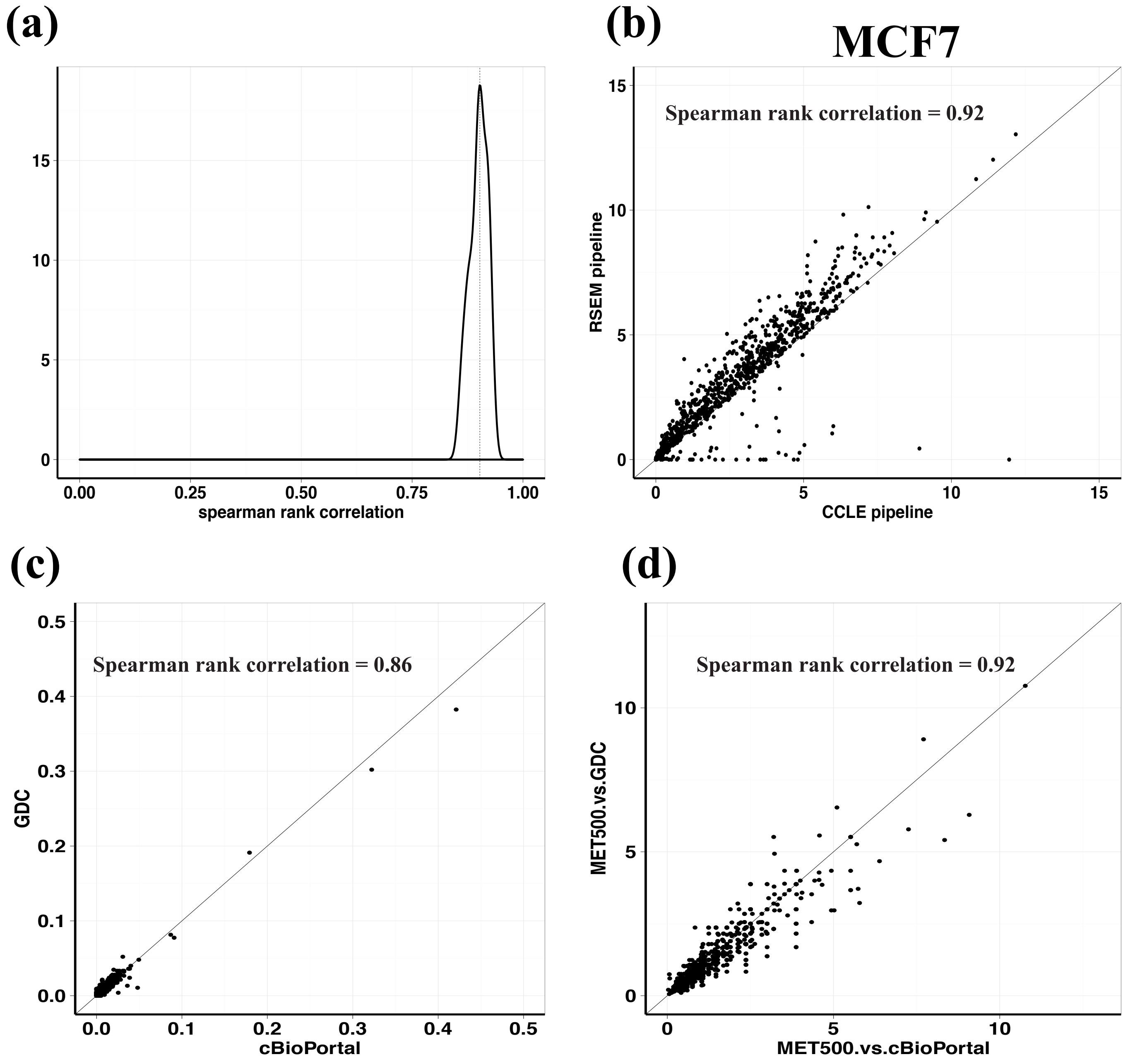

Supplementary Figure 11. (a) The density plot of spearman rank correlation values computed for 55 CCLE breast cancer cell lines. The vertical dashed line indicates median value of the distribution 0.9. (b) Gene expression values quantified by two different pipelines are highly correlated in MCF7. Each dot is a gene, x-axis represents its expression value quantified by CCLE pipeline and y-axis represents its expression value quantified by RSEM pipeline. (c) Gene mutation frequency values are highly correlated between cBioPortal and GDC. Each dot is a gene, x-axis represents its mutation frequency (across TCGA Breast Invasive Ductal Carcinoma cohorts) in cBioPortal database and y-axis represents its mutation frequency in GDC database. The solid line represents y=x. (d) P-values of gene differential-mutation analysis were highly correlated between cBioPortal and GDC. Each dot is a gene, x-axis represents its differential mutation P-value (in -log10 scale) derived from MET500-vs-cBioportal comparison and y-axis represents that derived from MET500-vs-GDC comparison. The solid line represents y=x.
