# Supplementary material for: Evaluating cell lines as models for metastatic breast cancer through integrative analysis of genomic data
Source: Nat Commun. 2019 May 15;10:2138. doi: 10.1038/s41467-019-10148-6 (PMC6520398; doi:10.1038/s41467-019-10148-6)
Supplement: Supplementary file 3 — Description of Additional Supplementary Files [file 41467_2019_10148_MOESM3_ESM.pdf]

Description of Additional Supplementary Information

File Name: Supplementary Data 1.
Description: Mutation frequency of the 75 highly (or differentially) mutated genes in CCLE, TCGA, and MET500 dataset.

File Name: Supplementary Data 2.
Description: Characteristics of the 57 CCLE breast cancer cell lines. Information in the first nine columns is from Table3 of Jiang's research paper.

File Name: Supplementary Data 3.
Description: P-value and adjusted FDR derived from subtype-specific TC analysis. Each row is associated with a CCLE breast cancer cell line.

File Name: Supplementary Data 4.
Description: Results of GO enrichment analysis.

File Name: Supplementary Data 5.
Description: Results of DA analysis.

File Name: Supplementary Data 6.
Description: Detailed statistics of the datasets used in our study.
